# Re-implantation of cryopreserved ovarian cortex resulting in restoration of ovarian function, natural conception and successful pregnancy after haematopoietic stem cell transplantation for Wilms tumour

**DOI:** 10.1007/s10815-016-0805-2

**Published:** 2016-09-17

**Authors:** C. E. Dunlop, B. M. Brady, M. McLaughlin, E. E. Telfer, J. White, F. Cowie, S. Zahra, W. H. B. Wallace, R. A. Anderson

**Affiliations:** 1 Simpson’s Centre for Reproductive Health, Royal Infirmary of Edinburgh, Edinburgh, EH16 4SA UK; 2 MRC Centre for Reproductive Health, University of Edinburgh, Edinburgh, EH16 4TJ UK; 3 Centre for Integrative Physiology, University of Edinburgh, Edinburgh, EH8 9XD UK; 4 Beatson West of Scotland Cancer Centre, Glasgow, G12 0YN UK; 5 Tissue and Cells Services, Scottish National Blood Transfusion Service, Edinburgh, EH17 7QT UK; 6 Royal Hospital for Sick Children, Edinburgh, EH9 1LF UK

**Keywords:** Ovarian cortex re-implantation, Fertility preservation, Premature ovarian insufficiency, AMH

## Abstract

With the improvement of long-term cancer survival rates, growing numbers of female survivors are suffering from treatment-related premature ovarian insufficiency (POI). Although pre-treatment embryo and oocyte storage are effective fertility preservation strategies, they are not possible for pre-pubertal girls or women who cannot delay treatment. In these cases, the only available treatment option is ovarian cortex cryopreservation and subsequent re-implantation. A 32-year-old woman had ovarian cortex cryopreserved 10 years previously before commencing high-dose chemotherapy and undergoing a haematopoietic stem cell transplant for recurrent adult Wilms tumour, which resulted in POI. She underwent laparoscopic orthotopic transplantation of cryopreserved ovarian cortex to the original site of biopsy on the left ovary. She ovulated at 15 and 29 weeks post-re-implantation with AMH detectable, then rising, from 21 weeks, and conceived naturally following the second ovulation. The pregnancy was uncomplicated and a healthy male infant was born by elective Caesarean section at 36^+4^ weeks gestation. This is the first report of ovarian cortex re-implantation in the UK. Despite the patient receiving low-risk chemotherapy prior to cryopreservation and the prolonged tissue storage duration, the re-implantation resulted in rapid restoration of ovarian function and natural conception with successful pregnancy.

## Introduction

The developments in cancer treatments over the last few decades have resulted in greatly increasing numbers of survivors. However, this has come at a cost, with many patients suffering long-term adverse effects of their life-saving treatment. The ovaries are particularly susceptible to damage by chemotherapy and radiotherapy with the type and dose of therapy and age of the patient influencing the extent of the injury [1, 2]. Loss of fertility is a major concern for young women facing potentially sterilising treatment [3]; one study found that almost three quarters of female cancer survivors of reproductive age have concerns regarding infertility and that survivors with irregular menses reported a lower quality of life than those with regular menstrual cycles [4]. Recent years have consequently seen significant developments in approaches to preserve fertility in those women and girls who are at risk of undergoing treatment-related premature ovarian insufficiency (POI) [5].

Embryo and oocyte cryopreservation are now established fertility preservation strategies [2, 5]; however, these are not appropriate in pre-pubertal girls, and in adult women the necessary ovarian stimulation may delay the start of cancer treatment. Ovarian tissue cryopreservation remains the only option in these situations. This approach was first validated in a large animal model [6] and has been offered to selected patients since 1993 [7]. The first human live birth as a result of ovarian cortex re-implantation was reported over a decade ago [8], and since then, there have been at least 60 live births described globally [9]. Yet, it is still regarded as an experimental technique with an uncertain success rate. We here describe the first human ovarian cortex re-implantation in the UK and report resumption of ovarian function resulting in a natural conception and pregnancy.

## Methods

**Fig. 1:**
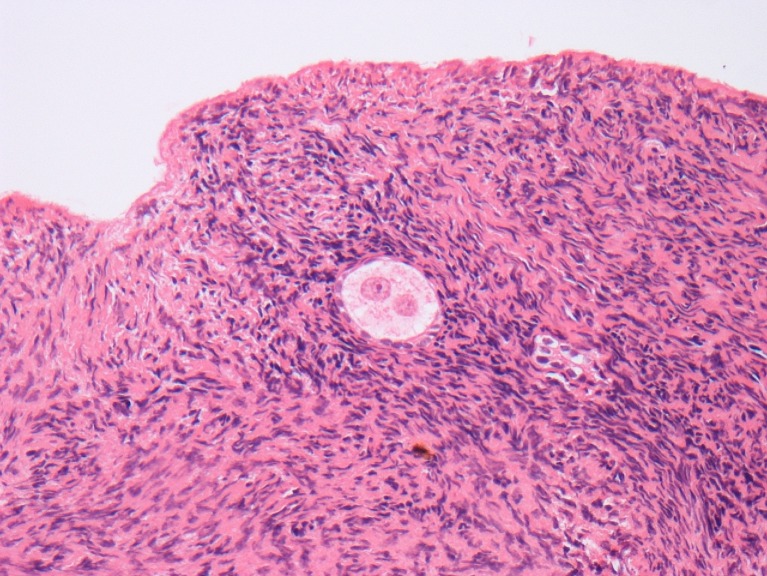
Biopsy of ovarian cortex removed at the time of ovarian cortex cryopreservation demonstrating a single primordial follicle with two nucleoli surrounded by normal ovarian stroma. The patient had already received non-sterilising chemotherapy prior to this biopsy

The patient was first referred to the Fertility Preservation service in Edinburgh, UK, at 22 years of age for discussion of ovarian cortex cryopreservation. She was a nulliparous, non-smoker with a normal body mass index and a past medical history of juvenile rheumatoid arthritis. She had presented with a right-sided abdominal mass at 21 years old and diagnosed with a stage 1 adult Wilms tumour of the right kidney. Following a right nephrectomy and adrenalectomy, she received adjuvant chemotherapy in the form of 5 cycles of vincristine and actinomycin D, following which her menstrual cycles continued. However, 3 months later, she had a recurrence of the tumour in the right renal bed, and a CT scan detected multiple pulmonary metastases. As the proposed treatment was deemed to be gonadotoxic, she was counselled regarding fertility preservation options. She elected for ovarian cortex cryopreservation, and 5 months following initial chemotherapy, she underwent laparoscopic removal of 18 biopsies of ovarian cortex from the left ovary with subsequent slow freezing [6] and long-term storage in the vapour phase of liquid nitrogen, with informed consent and ethical committee approval of the protocol. A piece of ovarian cortex sent for histopathological analysis showed the presence of a single primordial follicle, a corpus albicans, normal ovarian stroma and no evidence of malignant contamination (Fig. 1).

Subsequently, she received curative treatment consisting of chemotherapy comprising carboplatin (area under the curve (AUC) 10; 3 doses), etoposide (200 mg/m^2^; 9 doses), cyclophosphamide (500 mg/m^2^; 12 doses) and doxorubicin (30 mg/m^2^; 6 doses), resection of the recurrent tumour including partial hepatectomy, an autologous peripheral blood stem cell transplant (PBSCT) with pre-conditioning using high-dose melphalan (200 mg/m^2^; 1 dose) and radiotherapy. She received radiotherapy to the whole of both lungs (with a boost to the residual tumour sites) and to the right flank. The lower (inferior) border of the flank radiotherapy was the inferior edge of L2, and it extended from midline to the right lateral abdominal wall. The total dose to this region was 3000 cGy with the estimated dose to the right ovary being 100 cGy. The uterus was not in the field of radiation. This was followed by bilateral lower lobe resection of both lungs, with pathology showing no residual disease. These treatments induced POI, which was diagnosed by the presence of amenorrhoea, elevated gonadotrophins (follicle stimulating hormone (FSH) >100 IU/L and LH 53.1 IU/L) and low estradiol (E_2_; <70 pmol/L). Similar hormone measurements were obtained on repeat sampling, and hormone replacement therapy was commenced in the form of a combined oral contraceptive pill (COCP).

**Fig. 2:**
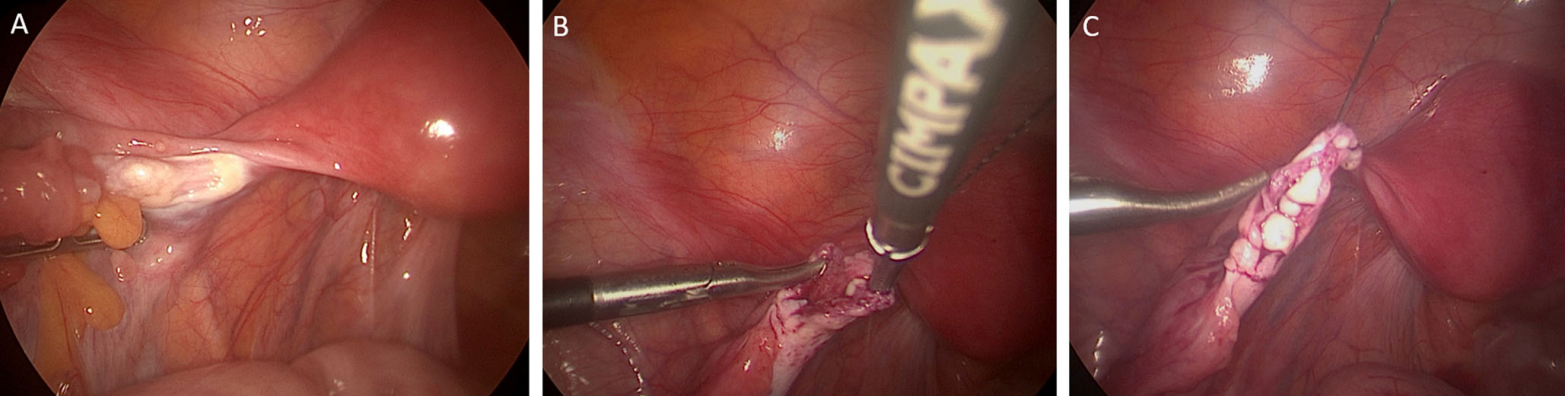
Laparoscopic orthotopic ovarian cortex re-implantation. **a** The atrophic native left ovary with scarring from previous ovarian cortical biopsy clearly visible. **b** A longitudinal incision was made along the left ovary resulting in a vascular surface. **c** Eight pieces of ovarian cortex were placed inside the native left ovary and adhered with fibrin sealant

At 32 years of age, the patient was disease-free and in a stable relationship, and requested re-implantation of her stored ovarian cortex with the aim of achieving a pregnancy. She had continued on COCP for hormone replacement in the interim. Given her medical history, she was reviewed in the pre-conceptual obstetric clinic and underwent cardio-respiratory assessment with an exercise tolerance test to assess her ability to carry a pregnancy. Pelvic ultrasound showed a normal uterus. Laparoscopic orthotopic transplantation of thawed ovarian cortex was performed in May 2015 (Fig. 2). The site of previous resection of ovarian cortex was clearly visible on the left ovary (Fig. 2a), with both ovaries atrophic but the pelvis otherwise normal with no adhesions. The tissue was thawed using a previously reported and validated protocol [6, 10]. A longitudinal incision along the left ovary was carried out and eight pieces of cortex (each approximately 1 cm × 1 cm × 0.5 cm) were then introduced onto the resultant cut ovarian surface, which was vascular but did not bleed actively. The tissue pieces were adhered to the ovary using fibrin sealant without suturing. The patient stopped her COCP at the time of the operation.

## Results

**Fig. 3:**
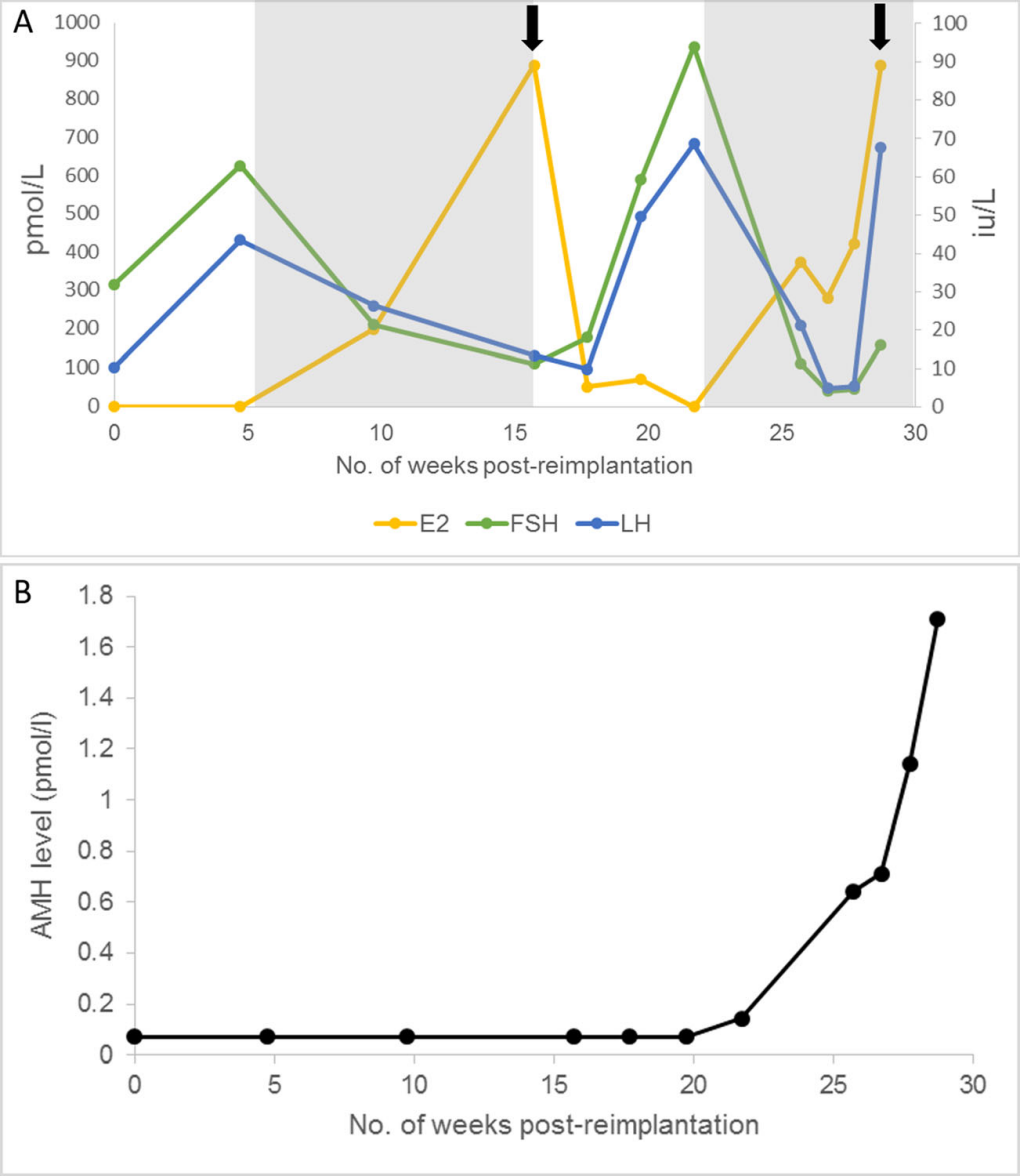
Serum hormones were measured following re-implantation, with day 0 being the day of re-implantation. **a** Serum gonadotrophin (follicle stimulating hormone (FSH), luteinising hormone (LH) and estradiol (E_2_) levels. The *grey boxes* illustrate time periods on hormone replacement therapy (HRT). Corresponding progesterone rises occurred following the two E_2_ peaks denoted by *black arrows*, confirming ovulation. **b** Serum anti-Mϋllerian hormone (AMH) levels rose steadily from 21 weeks post-re-implantation

**Fig. 4:**
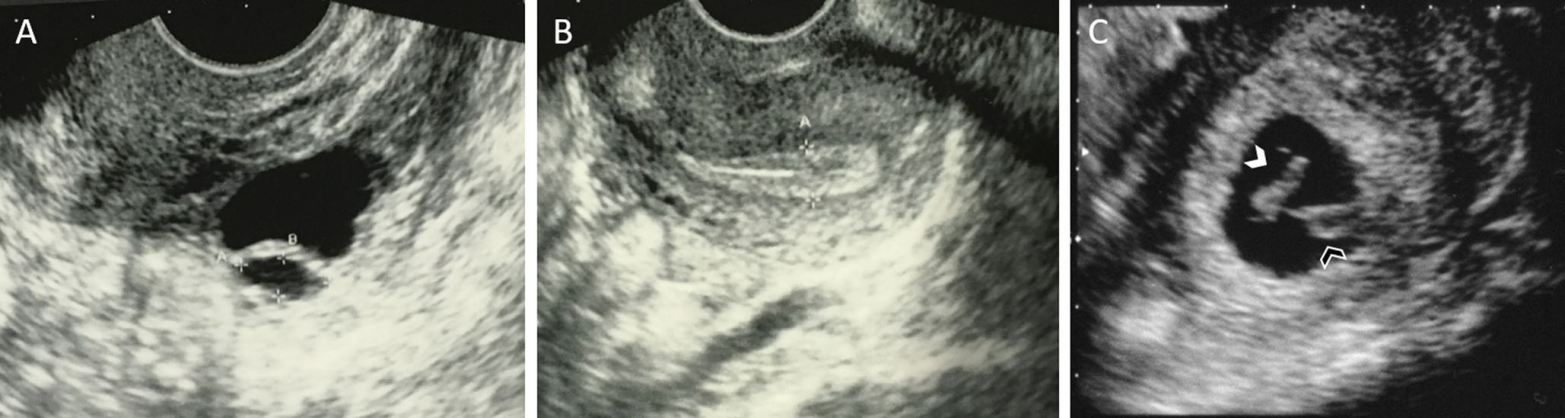
Transvaginal ultrasound scan images post-re-implantation. **a** Two antral follicles on the left ovary seen at 29 weeks postoperatively. The larger follicle had a mean diameter of 15 mm and ovulation was confirmed by a progesterone rise 1 week later. **b** Longitudinal view of the uterus at 29 weeks post-re-implantation demonstrating an endometrial thickness of 7.9 mm. **c** Single viable intrauterine pregnancy at 7^+3^ weeks gestation by crown-rump length (CRL) measurement (*white arrow*). The yolk sac is denoted by the *black arrow*. A corpus luteum was seen on the left ovary

The patient was followed up post-operatively with regular transvaginal ultrasound scans and serial measurement of serum gonadotrophins, E_2_ and anti-Mϋllerian hormone (AMH) (Fig. 3). Serum AMH was measured using an automated assay (Roche Elecsys, sensitivity 0.07 pmol/L). Due to problematic climacteric symptoms, low-dose hormone replacement therapy was recommenced 5 weeks postoperatively, with 1 mg oral E_2_ daily. Her gonadotrophins fell, and a follicle of mean diameter 14 mm on the left ovary was observed 15 weeks postoperatively, at which time FSH was 11.2 IU/L, LH 13.4 IU/L and E_2_ 891 pmol/L. She subsequently ovulated spontaneously with a confirmatory serum progesterone of 27.9 nmol/L 7 days later and menses a further week later. Hormone replacement therapy (HRT) was discontinued, but her menopausal symptoms returned; therefore, it was restarted 6 weeks later. She had a further spontaneous ovulation at 29 weeks postoperatively (peak E_2_ 891 pmol/L followed by progesterone 34.9 nmol/L) (Fig. 4a and b). Natural conception occurred, and a single, viable intrauterine pregnancy was confirmed on transvaginal ultrasound scanning at 7 weeks gestation (Fig. 4c), with a corpus luteum visible on the left ovary. AMH became detectable at 21 weeks post-re-implantation and rose progressively thereafter, although remaining low.

The pregnancy proceeded routinely, with normal ultrasound scans at 12, 20 and 28 weeks gestation. An elective Caesarean section was performed for maternal reasons (largely related to shortness of breath) at 36^+4^ weeks gestation with the delivery of a healthy male infant weighing 3.01 kg. Apgar scores were 9 at 1 min and 9 at 5 min, and cord blood gases were normal.

## Discussion

In this case report, we describe the first incidence of ovarian cortex re-implantation with natural conception and successful pregnancy in the UK. The ovarian tissue had been exposed to non-sterilising chemotherapy, had been cryopreserved for 10 years and demonstrated an apparent reduced ovarian reserve on pathology. Yet, ovulation was detected within 15 weeks of the re-implantation, and a viable pregnancy was achieved after the second ovulation. That the pregnancy has occurred from a follicle within the grafted tissue appears highly likely due to the patient suffering POI, diagnosed clinically and biochemically, for 9 years prior to re-implantation, the presence of a dominant follicle at the site of re-implantation and a corresponding corpus luteum following ovulation.

Although the patient reported initial improvement of menopausal symptoms in the weeks following replacement, HRT was later required for symptom relief, and it is notable that both ovulations occurred whilst taking oestrogen-only HRT. Oestrogen replacement increased the ovulation rate (and possibly pregnancy rate) in a randomised placebo-controlled trial in women with POI [11], with the effect confined to those with lower FSH concentrations during oestrogen treatment, probably reflecting less complete cessation of ovarian activity. Further RCTs are required to investigate the efficacy of this approach. Serum AMH remained undetectable following re-implantation for 20 weeks using a highly sensitive assay with limit of detection 0.07 pmol/L (0.01 ng/mL), then rose steadily over the next months until pregnancy. Thus, AMH remained undetectable until after the first ovulation but was thereafter consistently detectable, albeit at very low levels with a concentration of 1.8 pmol/L (0.25 ng/mL) at the time of conception. Using older assays, AMH has previously been reported to be generally undetectable post-re-implantation and therefore has not been considered valuable in monitoring ovarian function [12–14]. The present result therefore indicates that AMH may be of value in assessing the duration of function of ovarian transplants but requires the use of new, sensitive assays, although it must be recognised that AMH is not predictive of fecundability in young women [15].

Our findings are in keeping with those of the other main centres where ovarian cortex cryopreservation is performed [9, 14, 16]. A study of 60 cases of re-implantation worldwide reported the restoration of ovarian function in 93 % of patients, with a correlation seen between resumption of menses and number of follicles in the re-implanted tissue [14], whilst a case series of 95 re-implantations performed by the FertiPROTEKT network reported ovarian activity at 1 year after replacement in 63 % of recipients with POI [16]. A period of 3.5 to 6.5 months is generally observed before FSH falls and E_2_ rises [14]. Conception rates of 29–33 % have been reported, with 75–82.5 % of these pregnancies resulting in a live birth [9, 16]. Furthermore, natural conception has occurred in more than half of the cases, as here, highlighting that assisted reproductive techniques are not necessary for most women [14]. An alternative approach is repeated cycles of ovarian stimulation and embryo cryopreservation [17] although this is very invasive and costly and therefore of limited application. The great majority of these pregnancies have occurred following slow freezing of the ovarian cortical pieces [9]; however, a recent study indicated that vitrification may have advantages [18] and, indeed, two pregnancies following re-implantation of vitrified ovarian cortex have been reported [19].

This case illustrates that cryopreserved ovarian tissue can be stored for long periods of time with maintenance of follicle growth potential and oocyte viability. The longest reported duration of storage resulting in pregnancy is 11 years, in a young woman with sickle cell anaemia whose ovarian tissue was cryopreserved aged 14 [20]. In the present case, she had previously received chemotherapy with vincristine and actinomycin D which have very low gonadotoxicity [21], but it is striking that pathological analysis of a biopsy of ovarian cortex showed the presence of only one primordial follicle despite her young age at that time. Assessment of ovarian reserve by ultrasound or serum AMH was not possible at that time. Furthermore, re-implantation of ovarian tissue results in loss of the majority of follicles within the tissue [22]. However, it is well recognised that the distribution of follicles within the ovary in not even [23–25].

Preceding chemotherapy may affect graft function. The time to first ovulation in this case was more rapid than is generally observed in patients who had received chemotherapy prior to cryopreservation (less than 4 months in this case vs. 5.5–6.5 months [14]). This may not be related to her previous treatment; the rate of follicle activation increases with age and falling primordial follicle pool. The effect of cytotoxic treatments on ovarian reserve is variable and often difficult to predict, particularly as chemotherapy regimens frequently comprise a combination of drug classes. The alkylating agents used in her subsequent treatment, such as cyclophosphamide and melphalan (which are likely to have contributed to our patient’s POI, following her treatment for recurrence), are among the most toxic classes of chemotherapeutic agents [1]. However, a recent detailed analysis of childhood cancer survivors treated with chemotherapy without any possible effect of radiotherapy on gonadal function showed a risk of loss of fertility only with the highest doses of alkylating agent in females [26]. Furthermore, women who undergo a bone marrow transplant, as in this patient, are at extremely high risk of POI due to the requirement for highly toxic pre-conditioning regimens [27]. Previous chemotherapy is therefore important to consider when planning any form of fertility preservation procedure, with recent therapy potentially resulting in reduced viability of oocytes and in the longer term resulting in loss of ovarian reserve. However, this case highlights the likely importance of the patient’s young age (22 years old at tissue storage) and thus oocyte quality over low ovarian reserve in predicting successful conception. Indeed, the FertiPROTEKT case series demonstrated that re-implantation surgery was more successful in those patients who had their tissue cryopreserved at younger ages [16].

With regard to the surgical approach to re-implantation, several orthotopic strategies have been used (reviewed in [14]). In this case, we performed a similar procedure to that described by Sanchez-Serrano et al. [28] whereby the cortical pieces were inserted into a remaining ovary next to the medulla and sealed in place with a fibrin sealant. The pieces can also be sutured to the medulla, or placed in a subcortical area, although this was not performed here due to very high tissue friability and the availability of highly effective fibrin sealant. Another technique, required if the patient has no remaining ovaries, is the placing of ovarian pieces into a peritoneal window, preferably a vascular area, with the use of a sealant to keep the pieces adhered to the pelvic wall. Heterotopic re-implantations have also been performed. This method of transplantation has some advantages over orthotopic transplantation, including easier access for monitoring of graft function (reviewed in [14]); however, only one pregnancy has been reported as a result of the use of a heterotopic site (where the anterior abdominal wall was used) [29].

The average lifespan of ovarian tissue grafts appears to be 4–5 years [14] with much longer duration being reported in some women [13]. Five women have delivered at least twice from one re-implantation procedure to date [9]. Long-term ovarian function, comprising both fertility and hormone production can therefore be achieved; the latter is increasingly recognised to be of importance in the light of long-term adverse health effects of POI [30]. In this patient, ovarian function will be monitored post-pregnancy to assess the duration of the grafts, and half the stored tissue remains cryopreserved allowing the possibility of one or more further replacements. This case therefore adds to the growing evidence that ovarian tissue cryopreservation and later re-implantation are an important component of the fertility preservation repertoire, with the potential for restoration of fertility without the need for IVF and expensive medical interventions, and with the additional possibility of provision of long-term hormone production.
